# Selective isotropic etching of SiO_2_ over Si_3_N_4_ using NF_3_/H_2_ remote plasma and methanol vapor

**DOI:** 10.1038/s41598-023-38359-4

**Published:** 2023-07-18

**Authors:** Hong Seong Gil, Doo San Kim, Yun Jong Jang, Dea Whan Kim, Hea In Kwon, Gyoung Chan Kim, Dong Woo Kim, Geun Young Yeom

**Affiliations:** 1 School of Advanced Materials Science and Engineering, Sungkyunkwan University, Suwon, 16419 Republic of Korea; 2 SKKU Advanced Institute of Nano Technology (SAINT), Sungkyunkwan University, Suwon, 16419 Republic of Korea; 3 Department of Semiconductor Display Engineering, Sungkyunkwan University, Suwon, 16419 Republic of Korea

**Keywords:** Materials science, Nanoscience and technology

## Abstract

In this study, an isotropic etching process of SiO_2_ selective to Si_3_N_4_ using NF_3_/H_2_/methanol chemistry was investigated. HF was formed using a NF_3_/H_2_ remote plasma, and in order to remove the F radicals, which induces spontaneous etching of Si-base material, methanol was injected outside the plasma discharge region. Through this process, etch products were formed on the surface of SiO_2_, and then the (NH_4_)_2_SiF_6_ was removed by following heating process. When the H and F radicals were abundant, the highest SiO_2_ etch per cycle (EPC) was obtained. And, the increase of H_2_ and methanol percentage in the gas chemistry increased the etch selectivity by decreasing the F radicals. The etch products such as (NH_4_)_2_SiF_6_ were formed on the surfaces of SiO_2_ and Si_3_N_4_ during the reaction step and no noticeable spontaneous etching by formation of SiF_4_ was observed. By optimized conditions, the etch selectivity of SiO_2_ over Si_3_N_4_ and poly Si higher than 50 and 20, respectively, was obtained while having SiO_2_ EPC of ~ 13 nm/cycle. It is believed that the cyclic process using NF_3_/H_2_ remote plasma and methanol followed by heating can be applied to the selective isotropic SiO_2_ etching of next generation 3D device fabrication.

## Introduction

SiO_2_ films have been widely used in Si-based semiconductor devices together with Si_3_N_4_ films due to their excellent interfacial properties with Si, excellent dielectric properties, and easy processing^1^. These materials will be continuously used in next generation 3D semiconductor devices such as gate all around-field effect transistor (GAA-FET), and 3D memory devices, and, for these devices, highly selective isotropic etching processes in addition to highly selective anisotropic etching processes are required^2,3^. Currently, for selective isotropic etching of SiO_2_ and Si_3_N_4_, wet etching processes using hydrofluoric acid (for etching of SiO_2_) and phosphoric acid (for etching of Si_3_N_4_) are used^4,5^. However, wet etching processes may not be applicable for fabrication of next-generation devices of 10 nm or less due to the issues such as pattern leaning and collapsing caused by unbalanced capillary forces during drying of rinse liquid. In addition, since the etch rate is fast, it is difficult to control the process, and the problems might occur such as surface chemical damage by the etchant during etch process^6–8^. Therefore, it is necessary to develop selective isotropic dry etching processes that may solve these problems.

For a long time, gas-based SiO_2_ etching processes have been applied to remove native SiO_2_ layer formed on silicon wafer and thin SiO_2_ layer at the bottom of contact hole and, to thin or remove SiO_2_ hardmask layer. For isotropic dry etching of SiO_2_ selective to Si_3_N_4_ and/or Si, a vapor process using HF vapor as HF source^9–14^ and plasma processes forming HF by discharging F-based gases such as NF_3_, SF_6_, CF_4_, OF_2_, etc. and H-based gases such as NH_3_, H_2_, H_2_O, etc. have been studied^15–21^. In these processes, since SiO_2_ is hardly etched by anhydrous HF alone, a solvent that ionizes HF to react with SiO_2_ is required, and the solvents such as H_2_O, alcohol, NH_3_, etc. have been used. By using H_2_O or alcohol as a solvent, SiO_2_ reacts with ionized HF (HF_2_^-^, HF, H^+^, and F^-^) in the solvent and forms volatile SiF_4_^4,5,9,13^. For NH_3_ as a solvent, SiO_2_ is transformed into (NH_4_)_2_SiF_6_ salt by the reaction of HF and NH_3_, and the formed (NH_4_)_2_SiF_6_ is decomposed into NH_3_ and SiF_4_ by heating at a temperature of 100 °C or higher^14–19^. Among these, the etching by HF ionization is known to be difficult to control the etch process because the etch rate is fast similar to the wet process, etch rate varies depending on the quality of the SiO_2_ film, and additional rinse process may be necessary due to residual etchant^10,11,14^. So, HF/NH_3_ vapor process and the NF_3_/NH_3_ plasma process are more widely used. However, ammonium salts such as NH_4_F, NH_4_HF_2_ formed in the process using NH_3_^15–19^ can be a source of contamination in the chamber because they exist in the form of solid powder at room temperature^22,23^. Therefore, to avoid direct reaction between NH_3_ and HF in gaseous or plasma state, alternate SiO_2_ dry etching processes have been investigated through reaction with NF_3_ gas after inducing NH_3_ formation through N_2_/H_2_ plasma^24^ or through the formation of (NH_4_)_2_SiF_6_ on SiO_2_ by sequential supply of NH_3_ gas after adsorption of HF from HF vapor or from plasmas generated with SF_6_/H_2_ or NF_3_/H_2_^25,26^.

The above processes still use NH_3_ gas or NH_3_ formed through plasma which might generate particles, therefore, in this study, selective isotropic SiO_2_ dry etching process not generating noticeable NH_3_, by using H_2_/NF_3_ plasma as HF source and methanol (CH_3_OH) vapor as a solvent, has been investigated. In order to minimize the generation of F radical during dissociation of NF_3_ in the plasma which induces spontaneous etching of Si-base material^27^, H_2_/NF_3_ gases were supplied to the remote plasma area and methanol were injected to the processing area located outside the plasma discharge region. And, dual grids separating the discharge area and the processing area were located to block the ions from the discharge area. Through a cyclic process, saturated etch products such as (NH_4_)_2_SiF_6_ were formed on the surface of SiO_2_, and then the etch products were removed by heating for precise control of etched SiO_2_ thickness. The SiO_2_ etch characteristics were measured according to process conditions such as gas ratio, pressure, temperature, and process time, and the etch selectivities over Si_3_N_4_ and poly Si were compared. The etch mechanism was investigated by analyzing plasmas, surface composition, etc.

## Methods

200 nm thick SiO_2_ deposited by low pressure chemical vapor deposition (LPCVD) on silicon wafers was used to measure the etch depth during the dry etching. 50 nm thick Si_3_N_4_ deposited on silicon wafers and 50 nm un-doped poly silicon deposited on silicon oxide by LPCVD were used to measure the etch selectivity over SiO_2_. In addition, trench patterned silicon wafers (80 nm top critical dimension (CD), 150 nm pitch, and 440 nm depth) sequentially deposited with 5 nm thick LPCVD-Si_3_N_4_ and 10 nm thick LPCVD-SiO_2_ sequentially were used to investigate the etching effect on patterned samples. The quality of the Si_3_N_4_ used in this experiment was examined by wet etching in a HF solution (HF:deionized water = 1:100, RT) and the wet etch rates of Si_3_N_4_ and SiO_2_ used in this experiment are shown in Supplementary Fig. S1. As shown in Fig. S1, the etch selectivity of SiO_2_ over Si_3_N_4_ was ~ 50.

**Figure 1:**
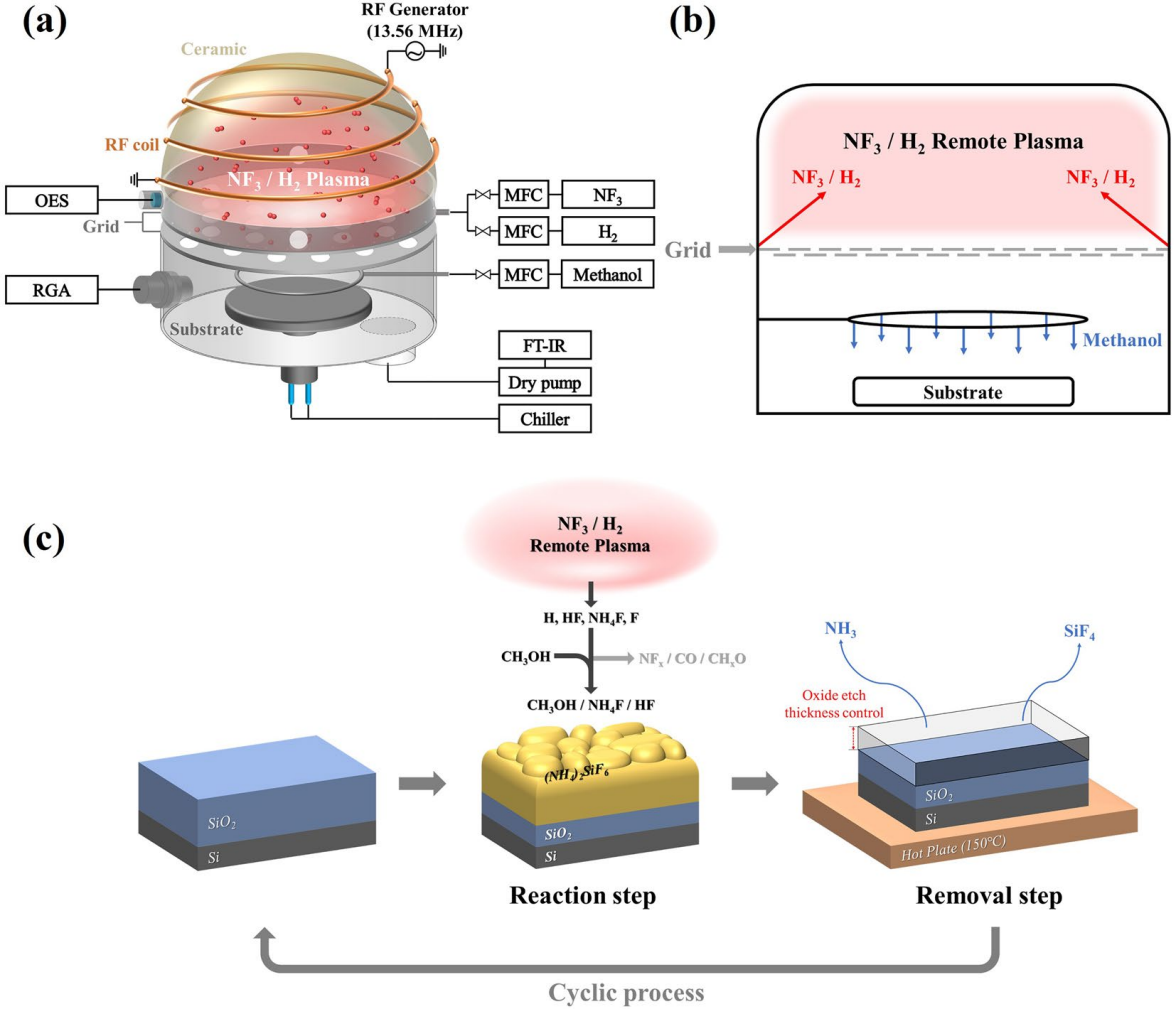
Schematic diagram of remote ICP type etcher and etch process steps. (**a**) Remote type ICP type etching system, (**b**) cross-sectional schematic diagram of the etcher with gas injections, and (**c**) cyclic etch process of SiO_2_.

The isotropic dry etching system used in this study is shown in Fig. 1a,b. NF_3_/H_2_ gas mixture was supplied to the plasma area, and the plasma was generated by an inductively coupled plasma (ICP) source operated with 13.56 MHz radio frequency (RF) power. Two anodized aluminum grids with crossed holes were located below the plasma source to block ions and flow radicals only to the processing area, and methanol gas was supplied to the substrate through a shower ring located between the substrate and grids. The substrate temperature was controlled using a bath circulator (JEIO TECH, HTRC-10) and the substrate temperature was measured using a thermocouple in contact with the wafer. The base pressure of chamber was maintained at ~ 1 × 10^–4^ Torr using a dry pump (ALCATEL ADIXEN, ADS 602P).

The etching process was consisted of a reaction step using remote plasma and a removal step using heating as shown in Fig. 1c. During the reaction step, reacted etch products were formed on the surface of SiO_2_ through radicals supplied from NF_3_/H_2_ plasmas through the grids and methanol vapor injected through the shower ring and, during the removal step, the formed etch products were removed by heating on a hot plate. For the reaction step, NF_3_/H_2_ plasma was formed with 500 W of RF power to the ICP source while maintaining the total operating pressure at 200 mTorr. Pressure ratios between NF_3_ and H_2_ gases in the plasma area and those between NF_3_/H_2_ gas mixture and methanol vapor were varied while keeping 200 mTorr. The substrate temperature was varied from 0 to 60 °C. For the removal step, formed etch products were removed by heating for 10 min. immediately after the plasma process on a hot plate (MTOPS, HSD180) preheated to 150 °C in a fume hood located next to the etcher. Also, the temperature and moisture of the etch room were controlled by using an air conditioning system.

Etch thicknesses of SiO_2_, Si_3_N_4_, and poly Si were measured using an ellipsometer (Nano-View SE MG-1000). To understand the etching mechanism, an optical fiber was connected to a quartz window located on the top of the grid as shown in Fig. 1a, and gas species generated in the plasma were analyzed using optical emission spectroscopy (OES; Avantes, AvaSpec-3648). Radicals and recombined gas species outside the discharge region were analyzed through a residual gas analyzer (RGA; SRS, RGA200) located at the processing area below the grids. Gas phase Fourier transform-infrared spectroscope (FT-IR; MIDAC corporation, I2001) was connected to the exhaust end of the dry pump and the gases generated during the reaction steps were analyzed at the exhaust area while a 5 × 5 cm^2^ sample located on the substrate holder was being processed. In addition, etch products formed on the blank samples and patterned samples were observed through field emission-scanning electron microscope (FE-SEM; HITACHI S-4700). Surface compositions and elemental bonding states of the SiO_2_ and Si_3_N_4_ after each step were analyzed through X-ray photoelectron spectroscopy (XPS; Thermo VG, MultiLab 2000, Al Kα source) after peak shift based on C 1 s (~ 285 eV) and through Fourier transform-infrared spectroscopy (FT-IR; Thermo Electron Nicolet 5700), respectively. Also, the surface roughness for each step was analyzed using an atomic force microscope (AFM; Park System XE-100).

## Results and discussion

**Figure 2:**
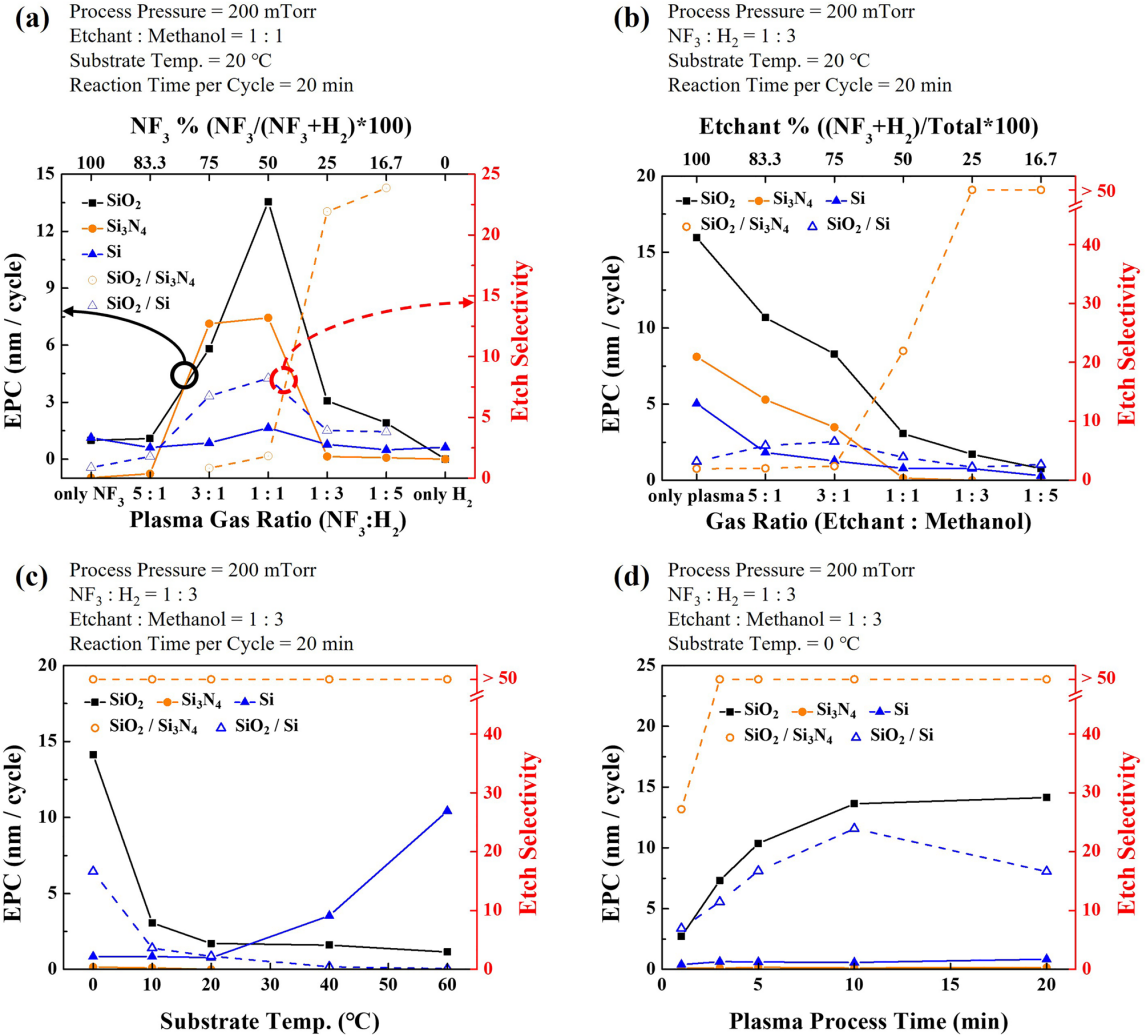
The EPC and etch selectivity of SiO_2_, Si_3_N_4_, and poly Si as a function of (**a**) NF_3_:H_2_ gas pressure ratio, (**b**) NF_3_/H_2_:methanol pressure ratio, (**c**) substrate temperature, and (**d**) process time of reaction step.

For the cyclic etching, SiO_2_, Si_3_N_4_, and poly Si were exposed to NF_3_/H_2_ remote plasma and methanol vapor for the formation of etch products (reaction step), and the etch products formed on the materials surface after the reaction were removed by heating at 150 °C for 10 min (removal step). The etch depth/cycle (EPC) of SiO_2_, Si_3_N_4_, and poly Si and their etch selectivities were measured as functions of various reaction step parameters and the results are shown in Fig. 2. Figure 2a shows the EPCs and etch selectivities measured a function of NF_3_/H_2_ gas ratio. The partial pressure ratios between NF_3_/H_2_ and methanol were kept at 1:1 for total operating pressure of 200 mTorr. The RF power, substrate temperature, and process time were maintained at 500 W, 20 °C, and 20 min, respectively. As shown in Fig. 2a, the SiO_2_ EPC was the highest at the ratio of NF_3_:H_2_ ~ 1:1 while the etch selectivity was increasing with increase of H_2_ in the NF_3_:H_2_. While keeping the ratio between NF_3_:H_2_ ~ 1:3, the partial pressure ratio between NF_3_/H_2_ and methanol was also varied and the results are shown in Fig. 2b. Other process conditions were the same as those in Fig. 2a. (The EPCs as a function of the different partial pressure ratios between NF_3_/H_2_ and methanol for different ratios of NF_3_:H_2_ are also shown in Supplementary Fig. S2). The increase of partial pressure ratios of methanol from 1:0 (NF_3_/H_2_ only) to 1:5 decreased etch rates of all materials but increased the etch selectivity of SiO_2_ over Si_3_N_4_ higher than 50 from the gas ratio of 1:5. With the ratio of NF_3_:H_2_ ~ 1:3 and the ratio of (NF_3_/H_2_): methanol ~ 1:3, the substrate temperature was varied from 0 to 60 °C while maintaining the other process conditions the same as Fig. 2a, and the etch results are shown in Fig. 2c. As shown in Fig. 2c, the decrease of substrate temperature increased EPC of SiO_2_ possibly due to the increased adsorption on the materials surfaces while keeping the etch selectivity of SiO_2_/Si_3_N_4_ higher than 50. When the plasma process time was varied for the optimized condition of the ratio of NF_3_:H_2_ ~ 1:3, the ratio of (NF_3_/H_2_):methanol ~ 1:3, and 0 °C, the SiO_2_ EPC was saturated after ~ 10 min while keeping the etch selectivity over Si_3_N_4_ higher than 50. In the case of etch selectivity of SiO_2_ over poly Si, the highest etch selectivity higher than 20 was also observed at the substrate temperature of 0 °C and the process time of 10 min for the ratio of NF_3_:H_2_ ~ 1:3 and the ratio of (NF_3_/H_2_): methanol ~ 1:3.

**Figure 3:**
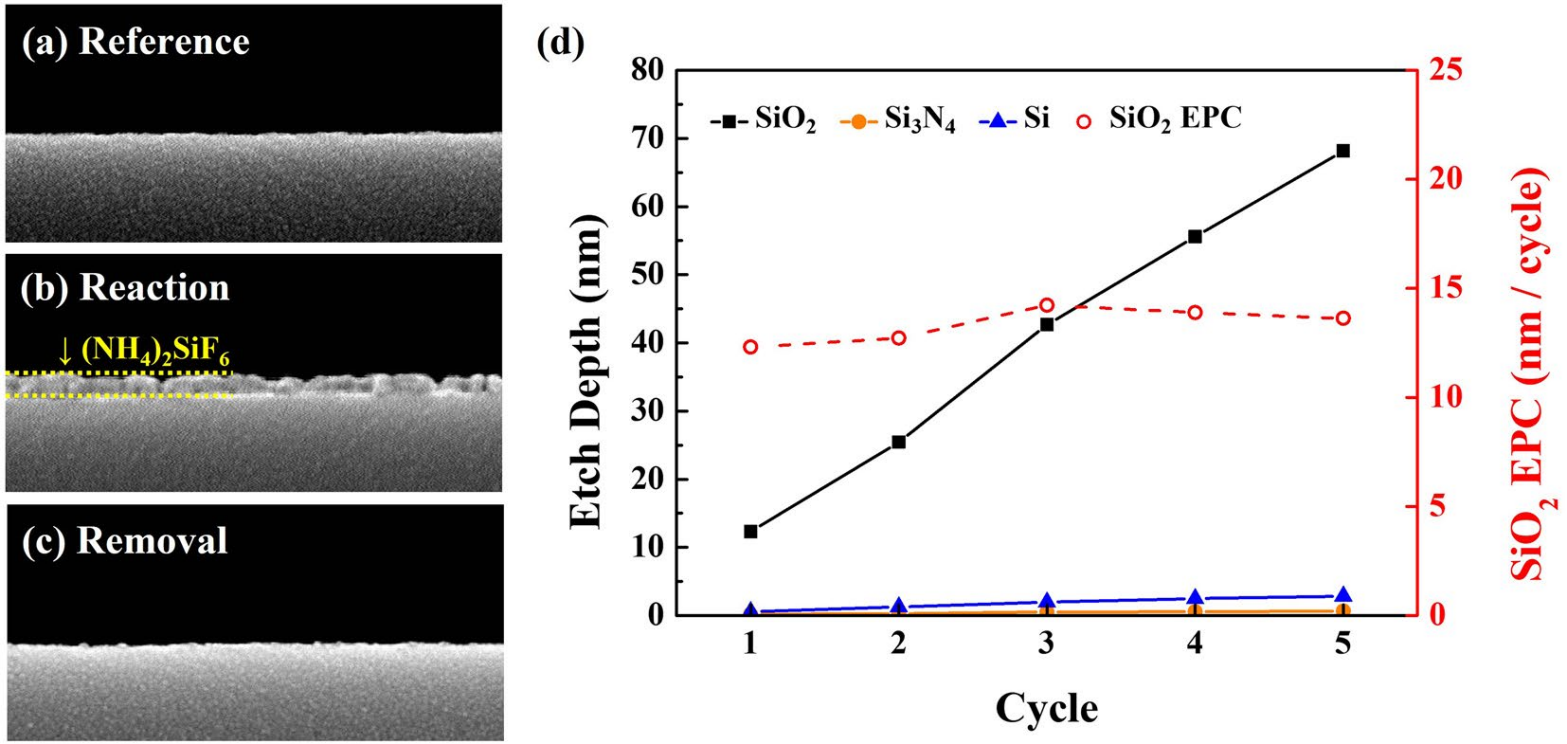
SEM images of SiO_2_ surface; (**a**) for reference, (**b**) after reaction step, and (**c**) after removal step. (**d**) Etch depth and EPC for 5 etch cycles. SiO_2_ surface was exposed to the optimized reaction conditions of NF_3_/H_2_ (1:3):methanol ~ 1:3, the substrate temperature of 0 °C, and process time of 10 min (reaction step), and the etch product formed on the surface was observed by SEM before and after the reaction, and after the removal by heating at 150 °C for 10 min using a hot plate (removal step).

SiO_2_ surface was exposed to the optimized reaction conditions of NF_3_/H_2_ (1:3):methanol ~ 1:3, the substrate temperature of 0 °C, and process time of 10 min (reaction step), and the etch products formed on the surface was observed by SEM before and after the reaction, and after the removal by heating at 150 °C for 10 min using a hot plate (removal step), and the results are shown in Fig. 3a–c. As shown in Fig. 3b, after the reaction step, etch products were observed on the SiO_2_ surface and, after the removal step, the etch products formed on the SiO_2_ surface were appeared to be removed. Tilted SEM images of the experimental results for Fig. 3b,c are shown in Fig. S3. While repeating the reaction and removal, the EPC and total etch depths were measured as a function of etch cycle and the result is shown in Fig. 3d. As shown in Fig. 3d, the EPC of SiO_2_ was similarly remaining at ~ 13 nm/cycle, therefore, the total etch depth was linearly increased with cycle number not only for SiO_2_ but also for all materials while keeping the SiO_2_ etch selectivity over Si_3_N_4_ over 50.

**Figure 4:**
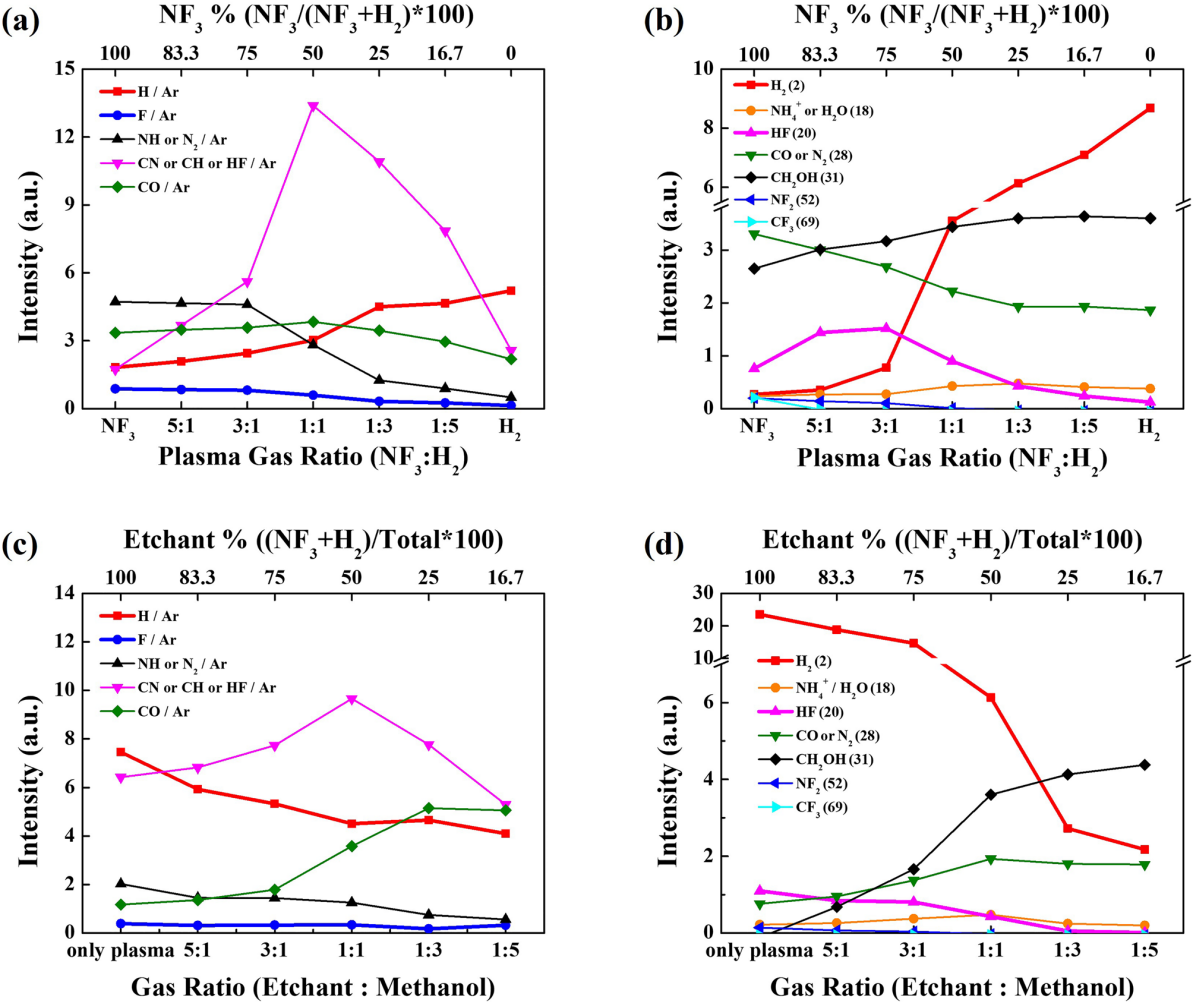
OES and RGA data as a function of (**a**,**b**) NF_3_:H_2_ pressure ratio and (**c**,**d**) NF_3_/H_2_:methanol pressure ratio. (**a**,**b**) are for the condition in Fig. 2a,c,d are for the condition in Fig. 2b.

To understand the EPC behavior observed in Fig. 2 as functions of various reaction parameters, the plasma characteristics in the plasma area and the species characteristics in reaction area under the grid were investigated using OES and RGA, respectively, and the results are shown in Fig. 4a,b for the condition in Figs. 2a and 4c,d for the condition in Fig. 2b. (OES spectra and RGA spectra can be found in Supplementary Figs. S4, S5 and S6, respectively) From OES, peaks related to H (656 nm), CO (519 nm), F (677 nm), NH or N (336 nm), and HF/CN/CH (388 nm) were observed, and, for the estimation of radical concentration, 10 mTorr of Ar was added during the OES measurement and the OES radical intensities were normalized by the Ar peak intensities at 750 nm. Figure 4a,c show the Ar normalized optical emission intensity ratios of species observed as a function of (a) NF_3_:H_2_ gas ratios and (c) NF_3_/H_2_:methanol ratios. As shown in Fig. 4a, with increasing H_2_ ratio, the increase of H intensity was observed while, with increasing NF_3_, the increase of F and N-related (NH or N_2_) peaks were observed due to the increase of related molecules in the gas mixture. Also, as shown in Fig. 4c, the increase of NF_3_/H_2_ in NF_3_/H_2_:methanol increased F, H, and N-related peaks while the increase of methanol in NF_3_/H_2_: methanol increased the CO also due to the increase of related molecules in the gas mixture. In addition, even though no hydrogen exists for NF_3_:H_2_ = 1:0 in Fig. 4a, H in addition to CO was detected in the plasma area and, as shown in Fig. 4c, even with decrease of H_2_ with increasing methanol, H did not decrease linearly possibly due to the formation of H through decomposition of methanol penetrated into the plasma area. Especially, as shown in Fig. 4a, c, the highest HF (or CN, CH but from Fig. 4b, it appears to be more related to HF) peak intensity was observed with the adequate mixture of NF_3_ and H_2_ possibly due to the highest formation HF from sufficient H and F in the plasma.

For RGA measured at the reaction area, masses such as H_2_ (2 amu), H_2_O (18 amu), HF (29 amu), CO or N_2_ (28 amu), CH_2_OH/CH_3_OH (31/32 amu), CF_3_ (69 amu), NF_3_ (71 amu), etc. were observed. However, highly reactive H (1 amu) and F (19 amu) radicals observed by OES in the plasma area were hardly measured in the reaction area by RGA due to recombination during the gas transportation from the plasma area to the reaction area. Figure 4b,d show the mass peak intensities observed as a function of (b) NF_3_:H_2_ gas ratios and (d) NF_3_/H_2_:methanol ratios for the conditions in Fig. 4a,c, respectively. As shown in Fig. 4b,d, with the increase of H_2_ in NF_3_:H_2_ and with the increase of NF_3_/H_2_ in NF_3_/H_2_:methanol, the increase of H_2_ was observed in the reaction area by RGA, while the increase of NF_3_ in NF_3_:H_2_ and the increase of methanol in NF_3_/H_2_:methanol increased CO/N_2_ possibly due to the reaction of CH_3_OH with F from NF_3_ and dissociation of CH_3_OH. HF molecules were observed by RGA in the reaction area as shown in Fig. 4b,d, and the HF molecules showed the highest peak with the adequate mixture of NF_3_:H_2_ at the reaction area and at the ratio of NF_3_/H_2_:methanol = 1:0 similar to the SiO_2_ etch results shown in Fig. 2a,b, respectively. Therefore, it is believed that, HF molecules formed by reaction of F with H (from H_2_ and decomposed CH_3_OH) in the plasma area and diffused to the reaction area are related to the SiO_2_ EPC observed in Fig. 2a,b.

**Figure 5:**
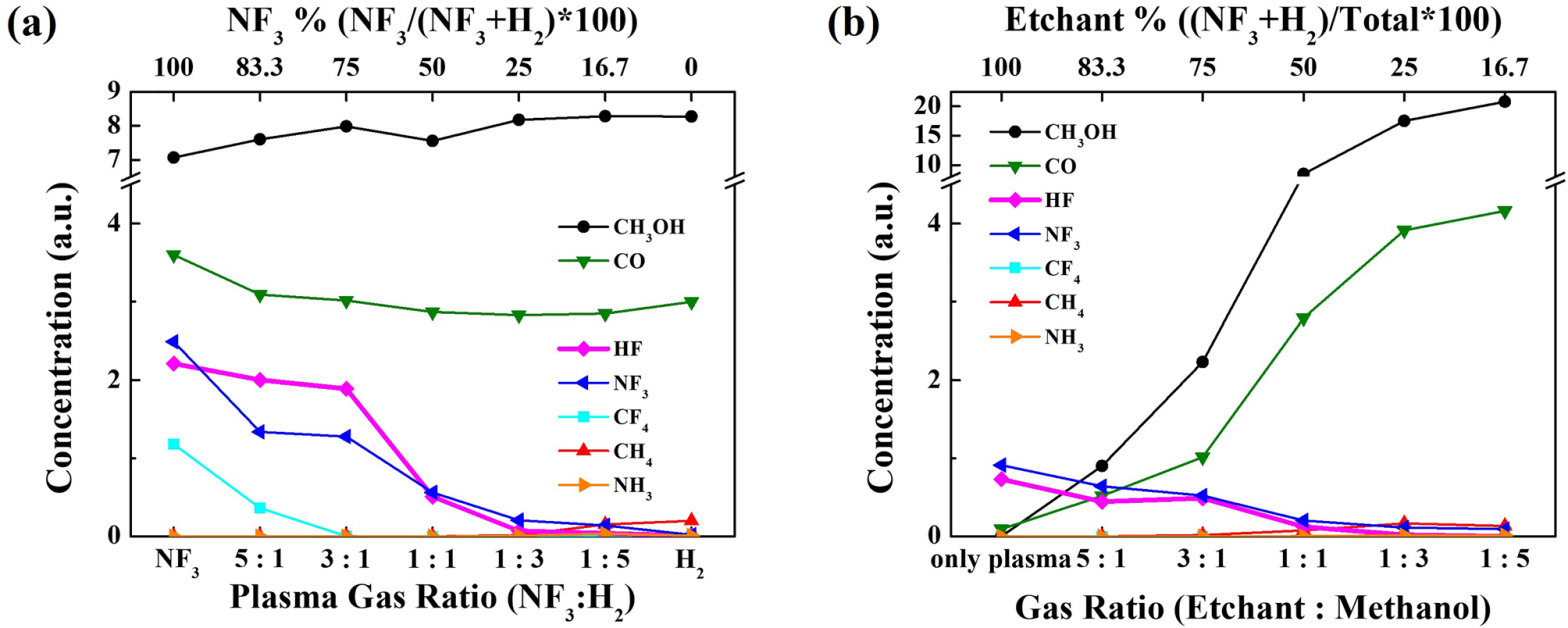
Gas phase FT-IR data measured as a function of (**a**) NF_3_:H_2_ pressure ratio and (**b**) NF_3_/H_2_:methanol pressure ratio for the process conditions in Fig. 2a,b, respectively.

While etching SiO_2_ with the conditions in Fig. 2a,b, the recombined species and etch products were observed using gas phase FT-IR located at the exhaust area. The species observed by gas phase FT-IR are shown in Fig. 5a for NF_3_:H_2_ gas ratios and (b) for NF_3_/H_2_: methanol ratios. Species such as CH_3_OH, CO, CH_4_, NF_3_, NH_3_, and HF were observed at the exhaust area by FT-IR. However, species such as H_2_, N_2_, O_2_ etc. could not be observed or measured by using FT-IR because these species do not absorb IR due to no changing dipole moments during the vibration that allow for the absorption of photons. As shown in Fig. 5, the variation of species observed by gas phase FT-IR with NF_3_:H_2_ gas ratios and NF_3_/H_2_:methanol ratios was similar to RGA data possibly due to the transport of recombined species without significant reaction from reaction area to the exhaust area. The FT-IR results obtained during the etching of Si_3_N_4_ and poly Si (not shown) under the same conditions as those obtained during the SiO_2_ etching were the same as Fig. 5a,b, and no SiF_4_ or SiH_4_ was also observed under all conditions. Therefore, it indicates that, during the reaction step, no noticeable spontaneous etching of SiO_2_, Si_3_N_4_, and poly Si with the reaction gases such as F and H in the reaction area was occurred.

**Figure 6:**
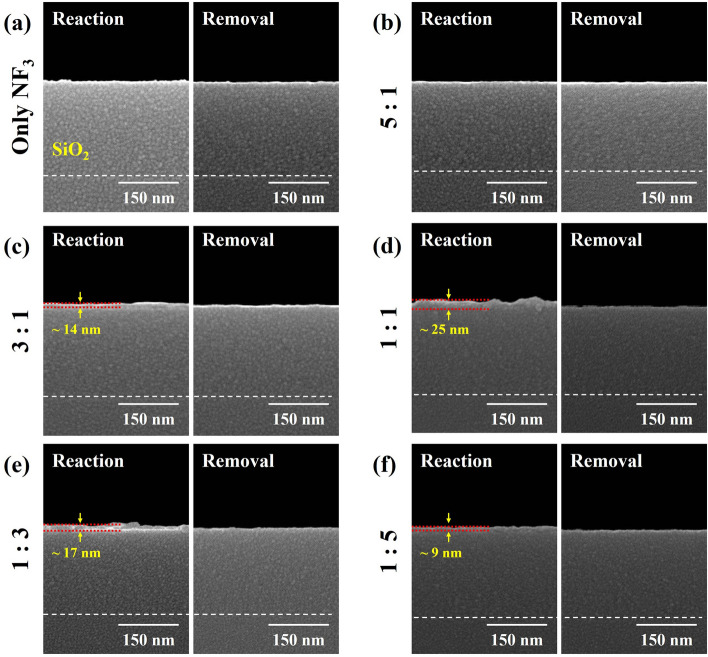
SEM images of SiO_2_ surface after reaction and removal steps according to NF_3_:H_2_ pressure ratio from NF_3_ only (1:0) to 1:5. The process conditions are same as those in Fig. 2a.

**Figure 7:**
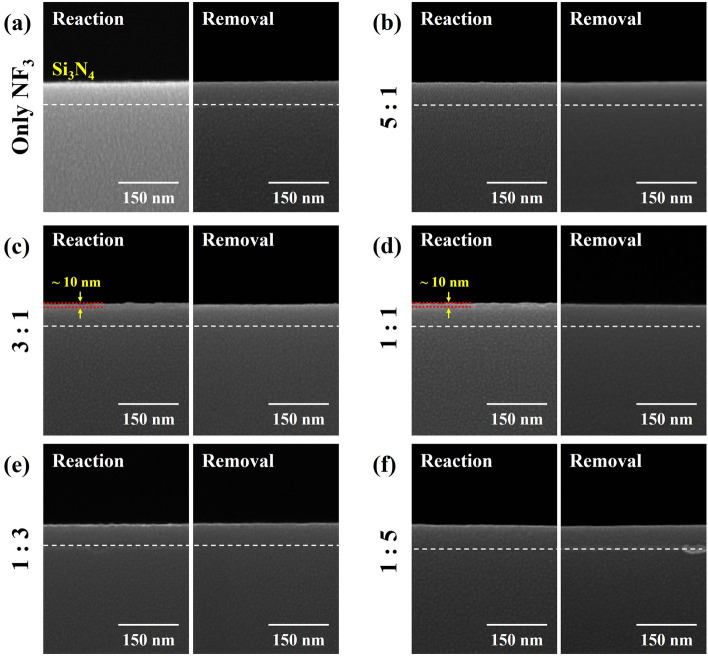
SEM images of Si_3_N_4_ surface after reaction and removal steps according to NF_3_:H_2_ pressure ratio from NF_3_ only (1:0) to 1:5. The process conditions are same as those in Fig. 2a.

The reaction compounds on the surfaces of SiO_2_ and Si_3_N_4_ after reaction step and removal step for the different NF_3_:H_2_ ratios were observed by SEM and the results are shown in Figs. 6 and 7 for SiO_2_ and Si_3_N_4_, respectively. The process conditions are the same as those in Fig. 2a. As shown in Fig. 6, after the reaction step, the reaction products formed on the surface were observed and, for high etch rate condition of NF_3_:H_2_ ~ 1:1, the thickest reaction product was observed. And, after the removal step, no noticeable reaction products on the SiO_2_ surface could be observed for all etch conditions. In case of Si_3_N_4_, as shown in Fig. 7, the thickest reaction product was also observed at the highest Si_3_N_4_ etch condition of NF_3_:H_2_ ~ 1:1, and after the removal step, no reaction products were observed on the Si_3_N_4_ surface for all etch conditions. Because no etch products such as SiF_4_ were observed during the reaction step as observed by gas phase FT-IR while forming the thickest reaction product at the condition of highest etch rate conditions, the reaction products were formed on the surfaces of Si_3_N_4_ and SiO_2_ during the reaction step without spontaneous etching of Si_3_N_4_ and SiO_2_ by F radicals and the reaction products were removed by heating during the removal step during the removal step. In fact, even with high F concentration for the condition of NF_3_:H_2_ = 1:0 as shown in Fig. 4a, no spontaneous etching of Si_3_N_4_ and SiO_2_ was observed as shown in Fig. 2a. From the data from OES, RGA, and gas phase FT-IR in Figs. 4 and 5, it is believed that F radicals formed from dissociation in the plasma area react with methanol (CH_3_OH) in the reaction area to form HF according to the formula below^28,29^;

1 $$ {\text{F }} + {\text{ CH}}_{{3}} {\text{OH }} \to {\text{ CH}}_{{2}} {\text{OH }} + {\text{ HF }}\left( { - {166}.{\text{8 kJ/mol}}} \right) $$

2 $$ {\text{F }} + {\text{ CH}}_{{3}} {\text{OH }} \to {\text{ CH}}_{{3}} {\text{O }} + {\text{ HF }}\left( { - {128}.{\text{7 kJ/mol}}} \right) $$

Therefore, no spontaneous etching of SiO_2_ and Si_3_N_4_ is appeared to be observed even with high concentration of F radicals in the plasma area. Some methanol also can participate in the discharge and is decomposed into H and CO, so it could act as H donor for HF formation for F radicals in the plasma area^30,31^. The increase of etch selectivity of SiO_2_ over Si_3_N_4_ obtained with the increase ratios of methanol in NF_3_/H_2_:methanol is also believed to be related to the formation of HF through the reaction of F with methanol.

**Figure 8:**
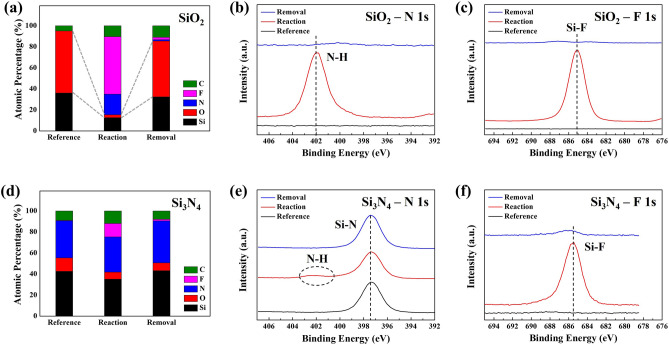
XPS analysis data for each process step under optimized conditions in Fig. 3; (**a**) atomic percentages of SiO_2_ surface for each process step, (**b**) N 1 s narrow scan of SiO_2_ surface, (**c**) F 1 s narrow scan of SiO_2_ surface, (**d**) atomic percentages of Si_3_N_4_ surface for each process step, (**e**) N 1 s narrow scan of Si_3_N_4_ surface, and (**f**) F 1 s narrow scan of Si_3_N_4_ surface.

To find out the materials characteristics of the reaction products formed on the surfaces of SiO_2_ and Si_3_N_4_, the surface characteristics were measured by XPS, FT-IR, and AFM. The process conditions are the same as those in Fig. 3. Figure 8 shows (a, d) atomic composition and narrow scan data of (b, e) N 1 s and (c, f) F 1 s for the surfaces of (a–c) SiO_2_ and (d–f) Si_3_N_4_ measured by XPS before (reference) and after reaction step, and after removal step with the optimized etch condition (XPS wide scan data of the surfaces of SiO_2_ and Si_3_N_4_ during the process steps are shown in Supplementary Fig. S7). As shown in Fig. 8a, for SiO_2_, after the reaction, the atomic percentages of Si and O were decreased from 36.1 to 12.7% and from 59.1 to 2.6%, respectively, while showing 54.7% of F after the reaction. The atomic percentage ratio between N and F in the etch product after the reaction was similar to (NH_4_)_2_SiF_6._ In the case of Si_3_N_4_, as shown in Fig. 8d, after the reaction, the atomic percentages of Si and N were slightly decreased from 42.6 to 35% and from 12.8 to 6.9% respectively, while showing 12.8% of F after the reaction due to the etch product formation similar to SiO_2_ even though the etch product thickness on Si_3_N_4_ was much thinner than that on SiO_2_. However, after the removal step, the surface compositions of both SiO_2_ and Si_3_N_4_ were almost recovered similar to those of references due to the removal of reaction products formed on the surfaces. Also, as shown in Fig. 8b,c,e,f, after the reaction step, the formation of etch products composed of N–H–Si–F could be identified for both SiO_2_ and Si_3_N_4_ by forming N–H bonding at 402 eV and Si–F bonding at 685 eV after the reaction step and, after the removal step, the removal of the etch products by removing those peaks. In addition, on the samples surfaces, ~ 10.9, 7.8% of carbon for SiO_2_ and Si_3_N_4_ and ~ 7.4% of oxygen for Si_3_N_4_ were continuously observed possibly due to the contamination and oxidation, respectively, due to the air exposure during the transport to XPS.

**Figure 9:**
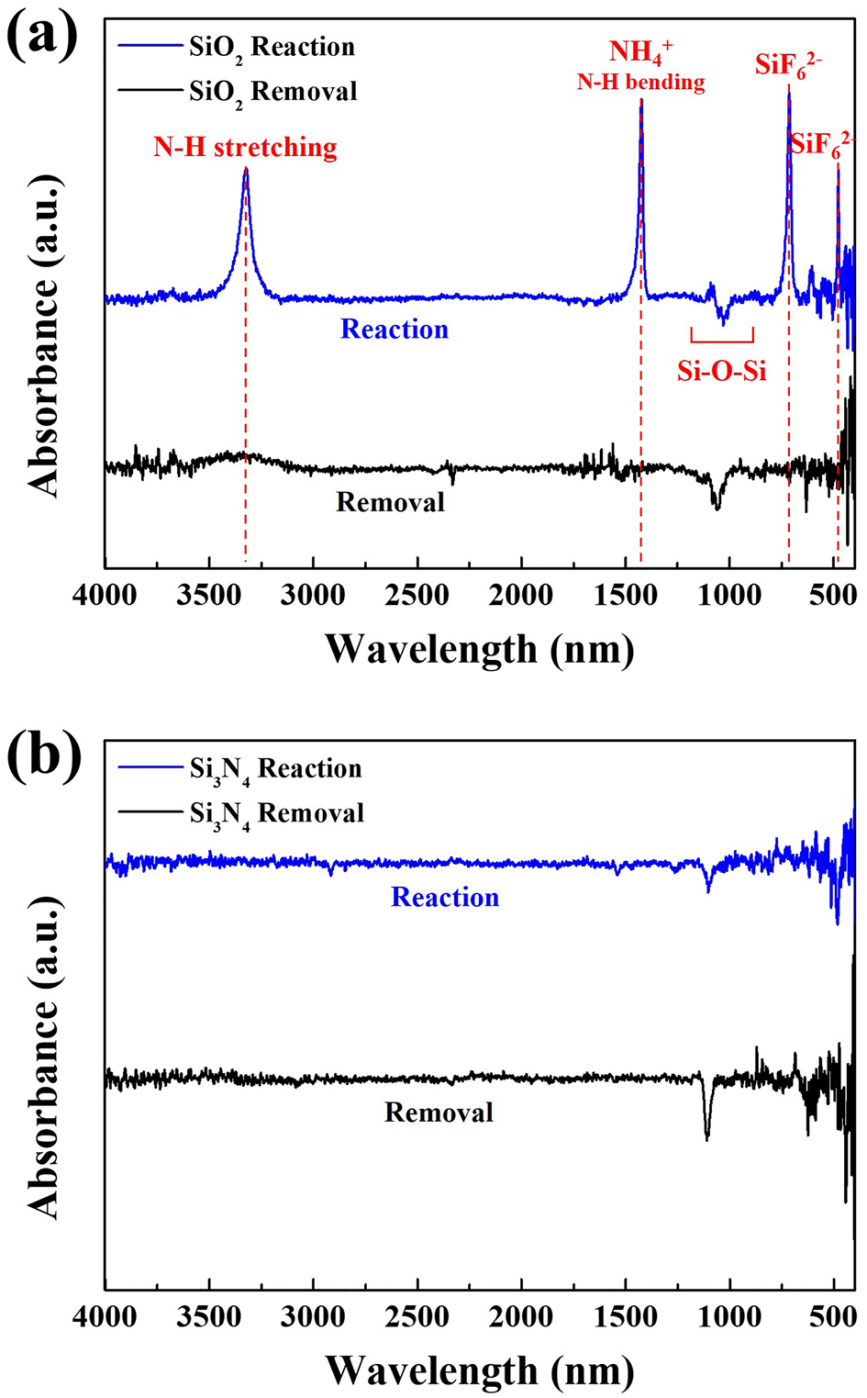
FT-IR analysis data for each process step under optimized conditions in Fig. 3; (**a**) FT-IR analysis result of SiO_2_ after reaction and removal steps and (**b**) FT-IR analysis result of Si_3_N_4_ after reaction and removal steps.

The binding states of etch products formed on the surfaces of SiO_2_ and Si_3_N_4_ after the reaction/removal steps using the optimized conditions of Fig. 3 were also observed using FT-IR and the results are shown in Fig. 9a,b, respectively. As shown in Fig. 9a, for SiO_2_, the absorption peaks such as N–H stretching (3330 cm^−1^), N–H bending (1454 cm^-1^) related to NH_4_^+^ bonding, and SiF_6_^2−^ bonding (717 cm^−1^, 478 cm^−1^) were observed after the reaction and, by comparing the XPS results in Fig. 8, it could be concluded that the etch products formed on the SiO_2_ surface after the reaction were related to (NH_4_)_2_SiF_6_. However, in case of Si_3_N_4_, as shown in Fig. 9b, possibly due to very thin etch product formation, no noticeable absorption peaks such as Si–F bonding related to etch products could be observed. In addition, after the removal steps, as shown in Fig. 9a,b, no absorption peaks could be also observed due to the removal of the etch product formed on the surfaces of both SiO_2_ and Si_3_N_4_. The XPS and FT-IR were also measured for poly Si and the results are shown in Supplementary Fig. S8. From the above observations, it is believed that the alkaline salt etch products such as (NH_4_)_2_SiF_6_ are formed by following reactions;

3 $$ {\text{2HF }} + {\text{ M }}\left( {{\text{solvent such as H}}_{{2}} {\text{O or Alcohol}}} \right) \, \to {\text{ HF}}_{{2}}^{-} + {\text{ MH}}^{ + } $$

4 $$ {\mathbf{For}} \, {\mathbf{SiO}}_{{\mathbf{2}}} :{\text{Si}} - {\text{O }} + {\text{ 2HF}}_{{2}}^{-} \to {\text{ SiF}}_{{4}} \left( {{\text{ads}}} \right) + {\text{ H}}_{{2}} {\text{O}}\left( {{\text{ads}}} \right), \,\,{\text{SiF}}_{{4}} + {\text{ 2NH}}_{{3}} + {\text{ 2HF}} \to \left( {{\text{NH}}_{{4}} } \right)_{{2}} {\text{SiF}}_{{6}} $$

5 $$ {\mathbf{For}} \, {\mathbf{Si}}_{{\mathbf{3}}} {\mathbf{N}}_{{\mathbf{4}}} :{\text{Si}} - {\text{N }} + {\text{ 2HF}}_{{2}}^{ - } + {\text{ MH}}^{ + } \to {\text{ SiF}}_{{4}} \left( {{\text{ads}}} \right) + {\text{NH}}_{{3}} \left( {{\text{ads}}} \right) \, + {\text{ M }} \to \, \left( {{\text{NH}}_{{4}} } \right)_{{2}} {\text{SiF}}_{{6}} + {\text{ M}} $$

In the above reactions, the formation of H_2_O for SiO_2_ etching and the formation of NH_3_ for Si_3_N_4_ etching can be important and the differences in etch rates, that is, differences in the etch product thickness could be related to the differences in the free energy of formation of H_2_O (− 265 kJ/mol) and NH_3_ (− 33.2 kJ/mol).

**Figure 10:**
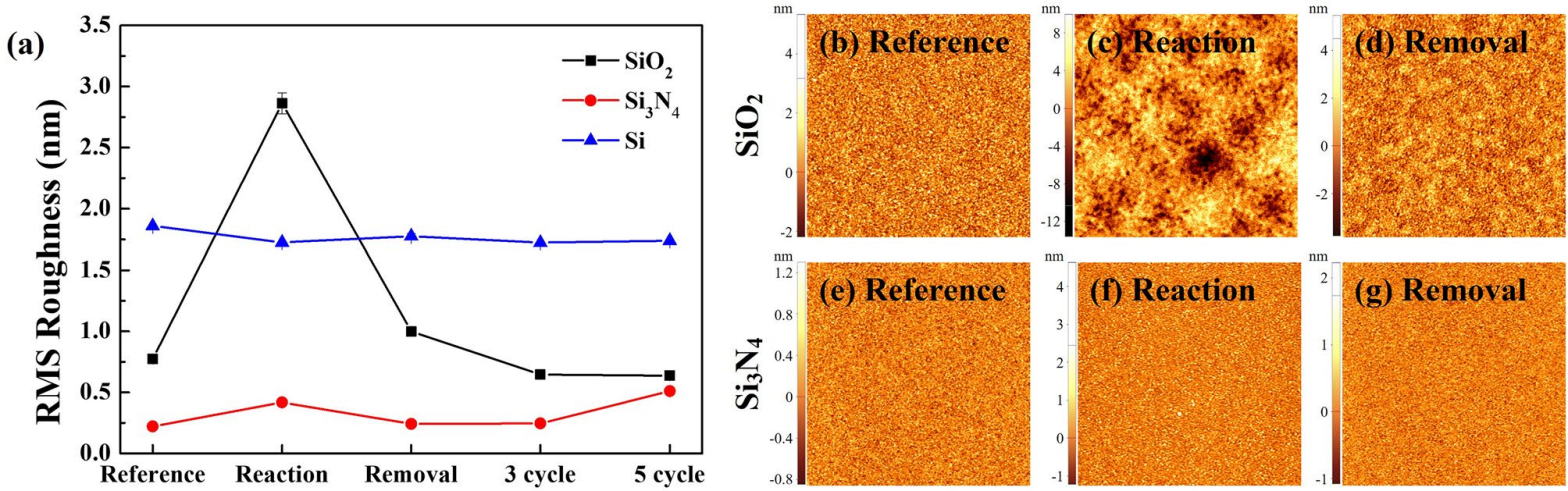
AFM analysis for each process step under optimized conditions in Fig. 3; (**a**) RMS roughness change of SiO_2_, Si_3_N_4_, and poly Si for 5 etch cycles. (**b**–**g**) AFM scan images of reference, after reaction step, and after removal step of SiO_2_ and Si_3_N_4_.

For the optimized conditions in Fig. 3, the change of surface roughness values during the each step of cyclic processing was observed using AFM and the RMS surface roughness values observed for SiO_2_, Si_3_N_4_, and poly Si are shown in Fig. 10a up to 5 cycles of etching and the 2D AFM surface roughness images of references, after reaction step, and after removal step for SiO_2_ and Si_3_N_4_ during 1st cycle of etching are shown in Fig. 10b–g. As shown in Fig. 10a,b–d, in the case of SiO_2_, after the reaction, the RMS surface roughness was significantly increased from 0.77 to 2.86 nm due to the formation of etch product such as (NH_4_)_2_SiF_6_. However, after the removal step, the RMS surface roughness was decreased to ~ 1.0 nm, and, even after 5 cycles of etching, the RMS surface roughness remained similar to that of reference. In the case of Si_3_N_4_, as shown in Fig. 10a,e–g, due to the very thin etch product formation during the reaction step, only slight increase of surface roughness from 0.22 to 0.42 was observed during the reaction step and, after the removal step, the RMS surface roughness was also decreased similar to that of the reference and no significant change was observed up to 5 cycles of etching. In the case of poly Si, possibly due to no formation of etch product similar to (NH_4_)_2_SiF_6_ during the reaction step, no significant change in RMS surface roughness was observed during the each step of etching. Therefore, on the surfaces of SiO_2_, Si_3_N_4_, and poly Si, no noticeable physical damage was observed by the cyclic etching.

**Figure 11:**
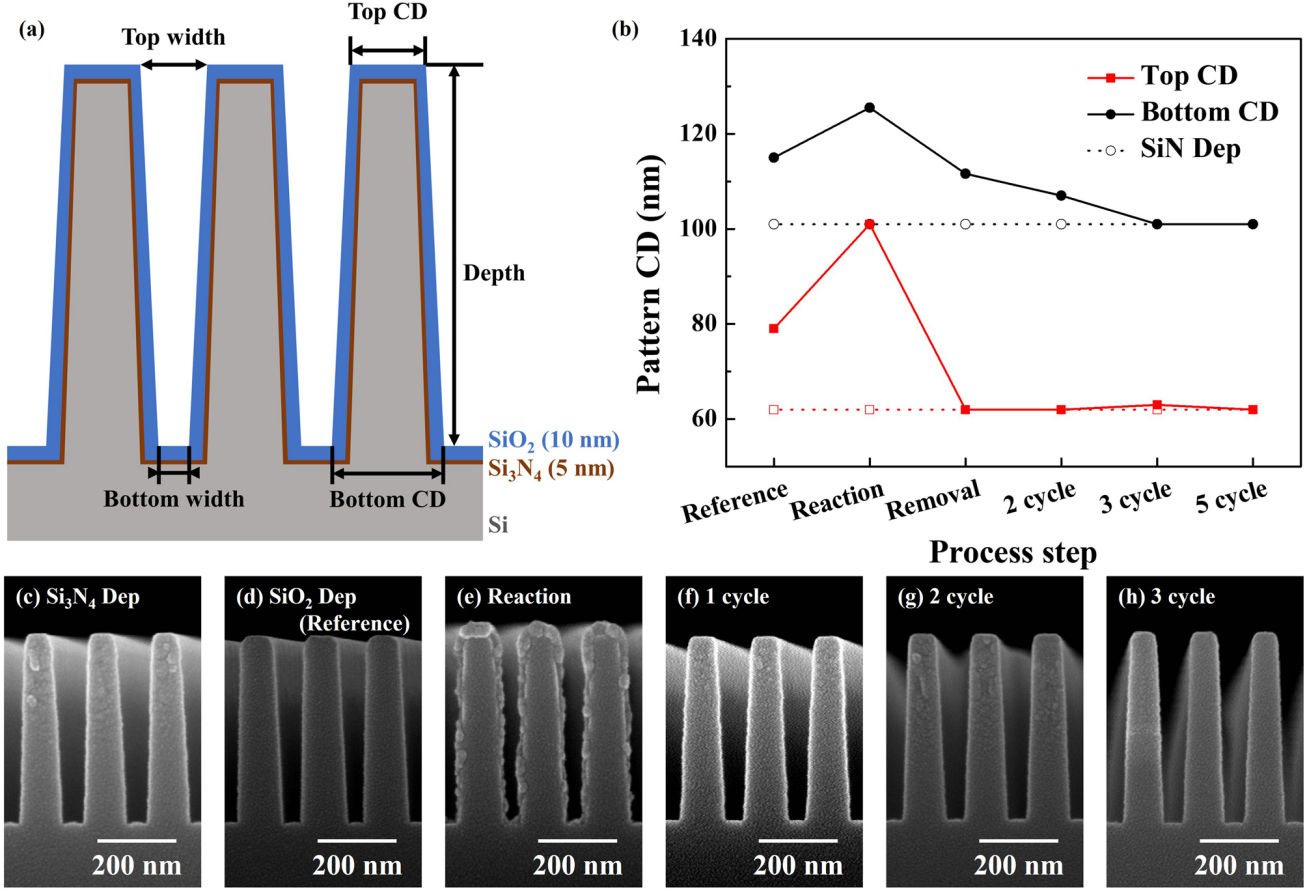
Etching of patterned silicon sample sequentially deposited with 5 nm thick Si_3_N_4_ and 10 nm thick SiO_2_ using the optimized etching conditions in Fig. 3; (**a**) schematic diagram of patterned sample reference, (**b**) CD change during process steps and cycles, and (**c**–**h**) SEM image of (**c**) after 5 nm Si_3_N_4_ deposition on Si trench, (**d**) after 5 nm Si_3_N_4_/10 nm SiO_2_ deposition (reference), (**e**) after reaction of 1st cycle, (**f**) after removal of 1st cycle, (**g**) after 2nd cycle, and (**h**) after 3rd cycle.

The optimized SiO_2_ cyclic etching selective to Si_3_N_4_ was applied to a patterned sample and the results are shown in Fig. 11. Figure 11a shows the schematic drawing of a patterned Si sample having the aspect ratio of ~ 10 and sequentially deposited with 5 nm thick Si_3_N_4_ and 10 nm thick SiO_2_. Actual SEM images of Si pattern after deposition of 5 nm thick Si_3_N_4_ and after following deposition of 10 nm thick SiO_2_ are shown in Fig. 11c,d, respectively. After the deposition of 5 nm thick Si_3_N_4_, the top/bottom CDs were ~ 60 nm/105 nm, and after the following deposition of 10 nm thick SiO_2_ (reference), the top/bottom CDs were changed to ~ 80 nm/115 nm. The Si trench pattern sample deposited with Si_3_N_4_/SiO_2_ was etched using the optimized condition in Fig. 3 and Fig. 11e,f show the SEM images after the reaction step and after the following removal step, respectively. As shown in Fig. 11e, after the reaction, due to the formation of reaction product, the top/bottom CDs were increased to ~ 100/125 nm, and the reaction product thickness was thicker on the top area (the byproduct thickness at the top of the trench was ~ 27.85 nm and that at the bottom of the trench was ~ 12.67 nm as shown in Fig. 11e). As shown in Fig. 11f, after the removal step, the etch product appeared to be removed and, the top/bottom CDs were decreased to ~ 60/111 nm. Due to the thinner etch product formation at the trench bottom area, three cycles were required to remove all SiO_2_ deposited on the Si pattern sample as shown in Fig. 11g,h. The measured top/bottom CDs are shown in Fig. 11b and, after the 3 cycles, the top/bottom CDs were the same as those of Si pattern deposited with 5 nm thick Si_3_N_4_, therefore, isotropic SiO_2_ etching selective to Si_3_N_4_ could be verified in a high aspect ratio patterned sample.

## Conclusions

In this study, isotropic dry etching of SiO_2_ selective to Si_3_N_4_ and poly Si was carried out using a two-step cyclic process composed of the etch products formation on SiO_2_ surface by using NF_3_/H_2_ remote plasma and methanol vapor, and the thermal desorption of the etch products by heat treatment. By using the cyclic process, ~ 13 nm/cycle of SiO_2_ etch per cycle (EPC) with the etch selectivity over Si_3_N_4_ higher than 50 for Si_3_N_4_ and that over poly Si higher than 20 could be obtained. The highest EPCs of SiO_2_ and Si_3_N_4_ were obtained when both H and F are abundant and the higher etch selectivity of SiO_2_ over Si_3_N_4_ could be obtained when H is richer than F in the system. Especially, methanol acted as H donor for HF formation for F radicals in the plasma area, therefore, the increase of methanol in the gas mixture increased the etch selectivity of SiO_2_ over Si_3_N_4_ even though the EPC of SiO_2_ decreased with increasing the methanol percentage. The etching of both SiO_2_ and Si_3_N_4_ was processed by formation and removal of etch products such as (NH_4_)_2_SiF_6_ not by forming volatile SiF_4_. When a silicon nanoscale trench patten sequentially deposited with Si_3_N_4_ and SiO_2_ was etched by the isotropic etching method, only SiO_2_ was etched on the nanoscale trench pattern by exposing silicon pattern surface deposited with Si_3_N_4_. Therefore, it is believed that this process can be applied for the selective isotropic etching of SiO_2_ for the next generation 3D devices, etc. Especially, by the formation of HF in the plasma without using the NH_3_ for the isotropic etching of SiO_2_, the formation of particles caused by NH_3_ is expected to be minimized.

## Supplementary Information

Supplementary Information.

## Data Availability

Data will be made available on request to Geun Young Yeom. (gyyeom@skku.edu).
