# Supplementary material for: Selective isotropic etching of SiO2 over Si3N4 using NF3/H2 remote plasma and methanol vapor
Source: Sci Rep. 2023 Jul 18;13:11599. doi: 10.1038/s41598-023-38359-4 (PMC10354158; doi:10.1038/s41598-023-38359-4)
Supplement: Supplementary file 1 — Supplementary Information. [file 41598_2023_38359_MOESM1_ESM.pdf]

Supplementary

Selective Isotropic Etching of SiO_2 over Si_3N_4 using NF_3/H_2 Remote Plasma and Methanol Vapor

Hong Seong Gil[1,=], Doo San Kim[1,=], Yun Jong Jang[1], Dea Whan Kim[3], Hea In Kwon[1], Gyoung Chan Kim[1], Dong Woo Kim[1,*], and Geun Young Yeom[1,2,*]

[1] School of Advanced Materials Science and Engineering, Sungkyunkwan University, Suwon 16419, Republic of Korea
[2] SKKU Advanced Institute of Nano Technology (SAINT), Sungkyunkwan University, Suwon 16419, Republic of Korea
[3] Department of Semiconductor Display Engineering, Sungkyunkwan University, Suwon 16419, Republic of Korea

=These authors contributed equally to this work.
*E-mail: dwkim111@gmail.com, gyyeom@skku.edu

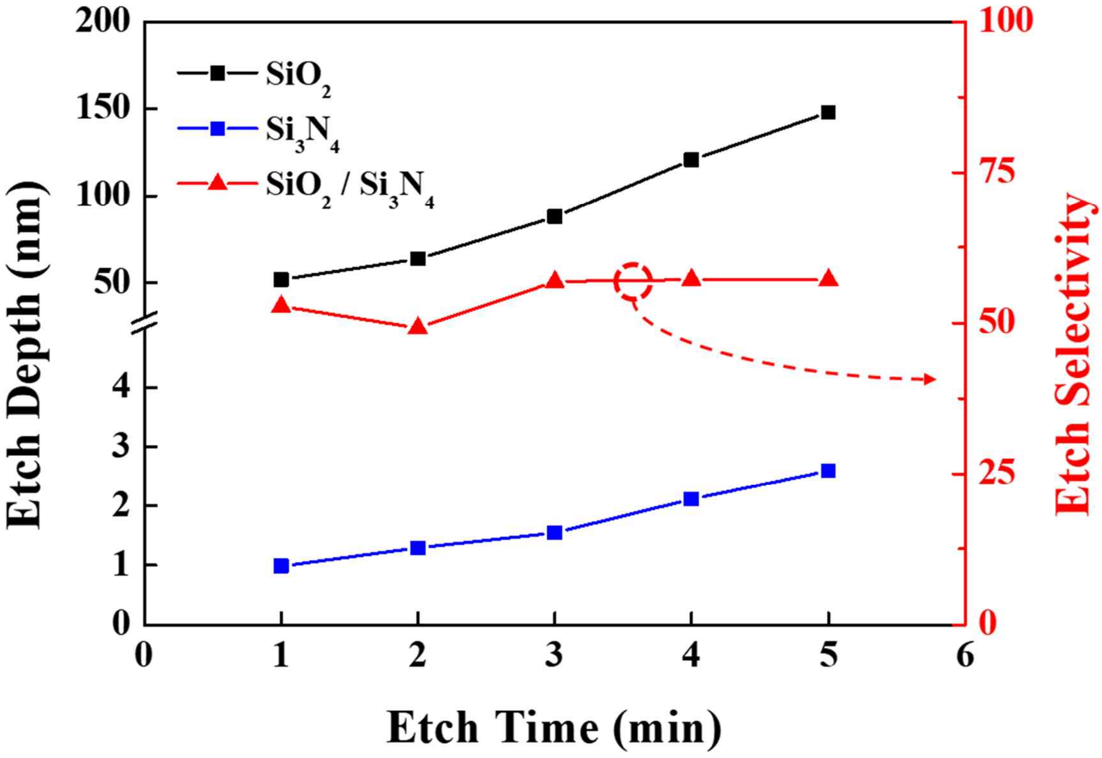

Figure S1. Wet etch rates of SiO_2 and Si_3N_4 samples used in the experiment and their etch selectivities by etching in a HF solution (HF:deionized water = 1:100, RT).

Figure S1 shows the etch rates of Si_3N_4 and SiO_2 used in this experiment with 1 % HF solution and, as shown in Figure S1, the etch rate of SiO_2 was ~ 30 nm/min and the etch selectivity of SiO_2 over Si_3N_4 was ~ 50.

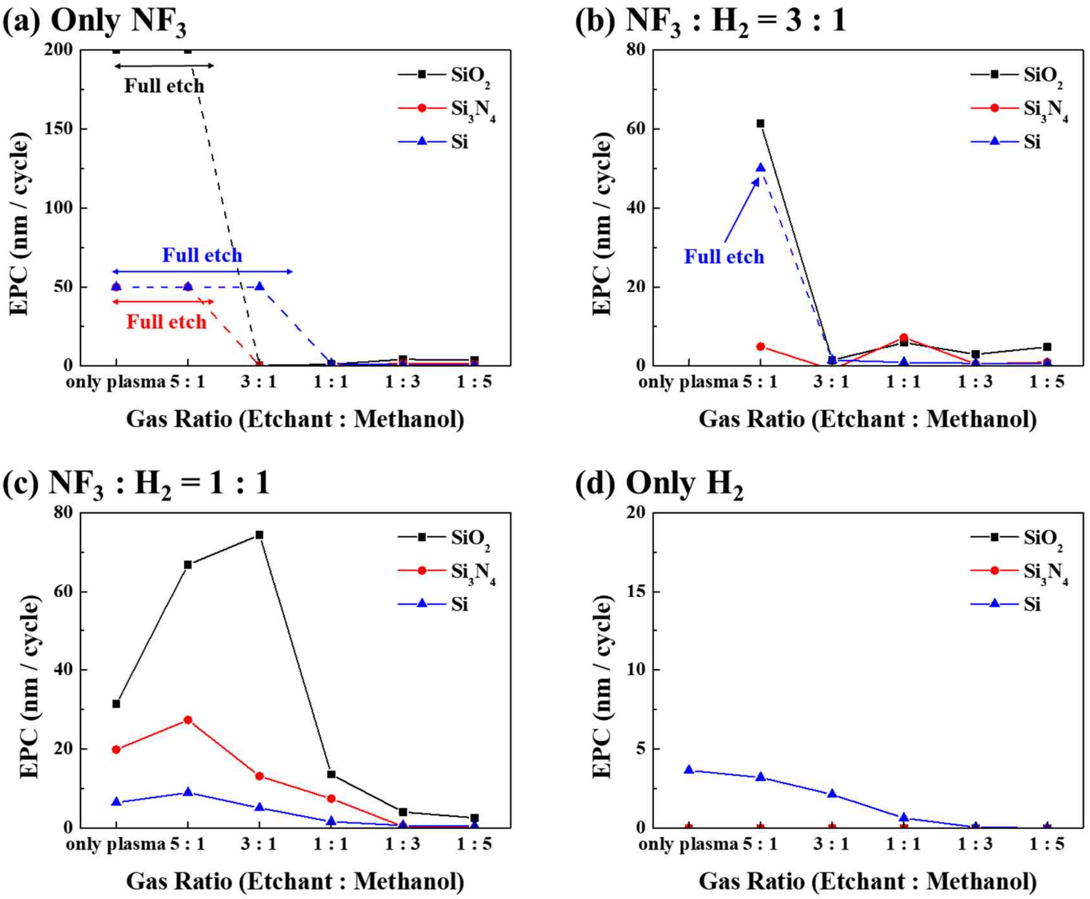

Figure S2. EPCs as a function of the partial pressure ratio between NF_3/H_2 and methanol for different ratios of NF_3 : H_2; (a) only NF_3 plasma, (b) NF_3 : H_2 ratio of 3 : 1, (c) NF_3 : H_2 ratio of 1 : 1, and (d) only H_2 plasma. Other process conditions are the same as those in Figure 2(b).

Figure S2 shows EPCs as a function of the partial pressure ratio between NF_3/H_2 and methanol for different ratios of $NF_3:H_2$. As shown in Figure S2(a), for NF_3 only, 200 nm thick SiO_2 and 50 nm thick Si_3N_4 and Si were etched away within one cycle when the ratio of NF_3:methanol was lower than 5:1, however, when the ratio of NF_3:methanol was higher, no etching was observed. Similar results were obtained for the NF_3-rich conditions such as $NF_3:H_2$ = 3:1 as shown in Figure S2(b). However, for the conditions of $NF_3:H_2$ = 1:1, the EPC was the highest when both NF_3/H_2 and methanol were abundant as shown in Figure S2(c). For the H_2 rich conditions, only silicon was etched as shown in Figure S2(d).

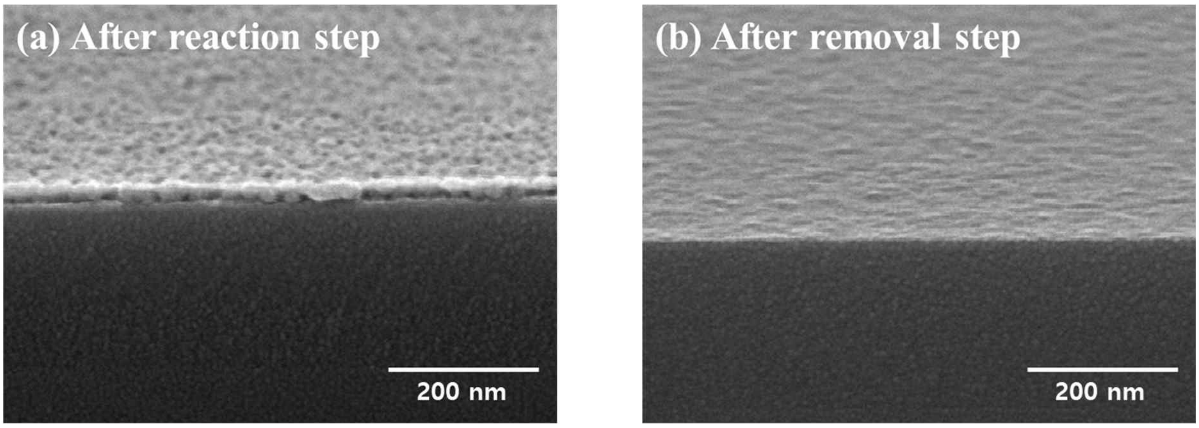

Figure S3. SEM tilt images of SiO_2 surface (a) after the reaction step and (b) after the removal step. SiO_2 surface was exposed to the optimized reaction conditions of NF_3/H_2 (1/3) : methanol ~ 1 : 3, the substrate temperature of 0 °C, and the process time of 10 min (reaction step). The etch products formed on the SiO_2 surface were observed by SEM (a) after the reaction and (b) after the removal of the reacted etch products by heating at 150 °C for 10 min using a hot plate.

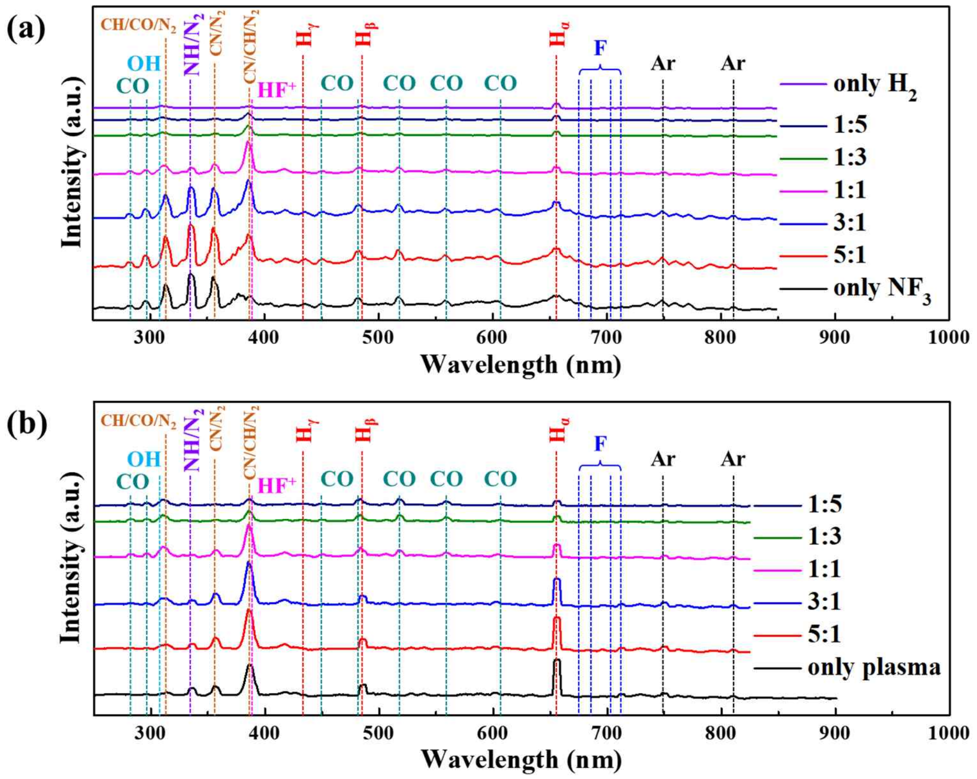

Figure S4. OES spectra in plasma region measured as a function of (a) NF_3 : H_2 gas ratio and (b) NF_3/H_2 : methanol ratio for the conditions in Figure 2(a) and (b), respectively.

Figure S3 shows the raw OES data in plasma region after the removal of backgrounds measured as a function of (a) NF_3:H_2 gas ratio and (b) NF_3/H_2:methanol ratio for Figure 4(a) and (b), respectively. For Ar actinometry, small Ar was added in the plasma and the Ar peaks could be measured at 750.4 nm and 811.5 nm. [1-6] Methanol related peaks were observed; OH peak at 309 nm [2,7], CO peaks at 297.7, 451, 483.5, 519.8, 559, 607 nm, etc. [4,8-10], NH peak at 336 nm, and CN peaks at 357, 388 nm. [4,6,8,9,11] N_2 peak was observed in the region of 300-400 nm, [4-8,11] H peaks at 434, 486, 656 nm, and F peaks at 677, 685.6, 703.7, 712.8 nm. [2-5,7] For Ar actinometry, Ar peak at 750 nm was used with H peak at 656 nm, F peak at 703.7 nm, NH at 336 nm, CN/CH at 388 nm, and CO at 519 nm.

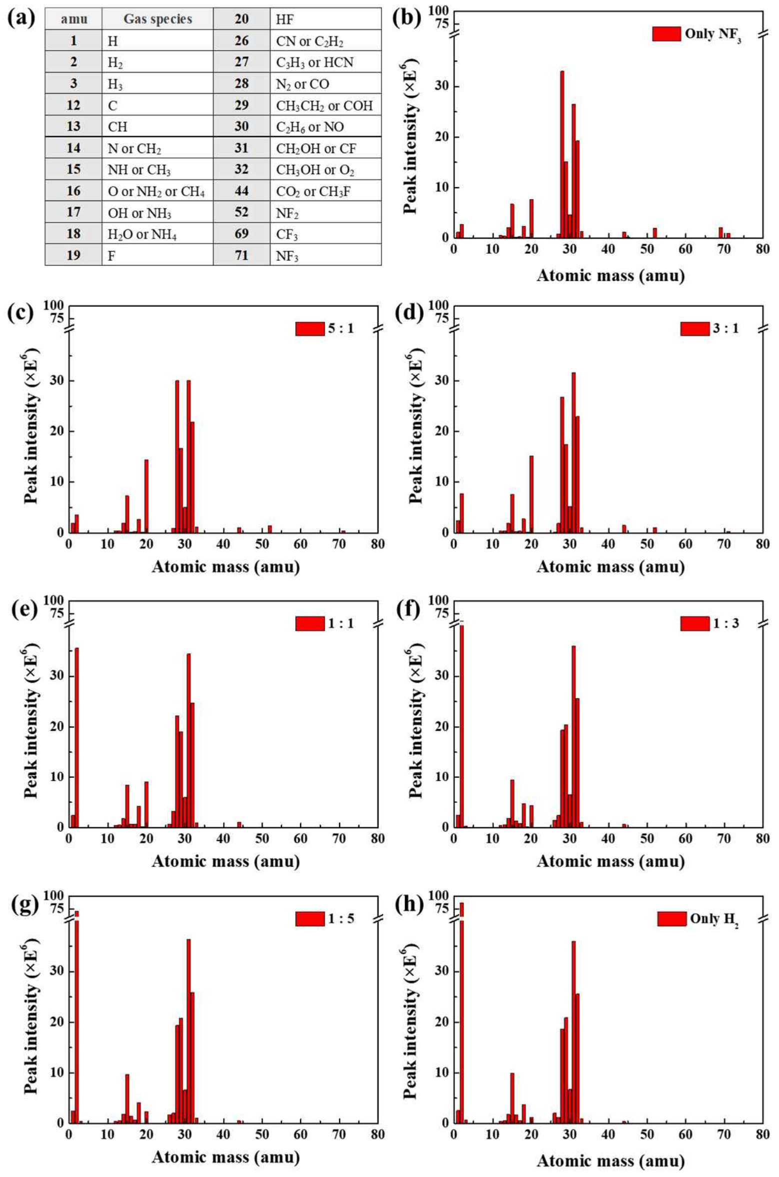

amu	Gas species	amu	Gas species
1	H	20	HF
2	H_2	26	CN or C_2H_2
3	H_3	27	C_3H_3 or HCN
12	C	28	N_2 or CO
13	CH	29	CH_3CH_2 or COH
14	N or CH_2	30	C_2H_6 or NO
15	NH or CH_3	31	CH_2OH or CF
16	O or NH_2 or CH_4	32	CH_3OH or O_2
17	OH or NH_3	44	CO_2 or CH_3F
18	H_2O or NH_4	52	NF_2
19	F	69	CF_3
		71	NF_3

Figure S5. RGA spectra in main chamber region for different NF_3 : H_2 gas ratios; (a) species according to amu, (b) only NF_3 condition, (c) 5 : 1 condition, (d) 3 : 1 condition, (e) 1 : 1 condition, (f) 1 : 3 condition, (g) 1 : 5 condition, and (h) only H_2 condition.

Figure S4 shows RGA spectra in main chamber region for different NF_3 : H_2 gas ratios. Atomic species such as H and F which could be observed by OES in the plasma region were not observed in the process chamber region possibly due to recombination. Also, possibly due to the ionization in the RGA, dissociated species such as CH_2OH, NF_2 representing CH_3OH, NF_3 were observed.

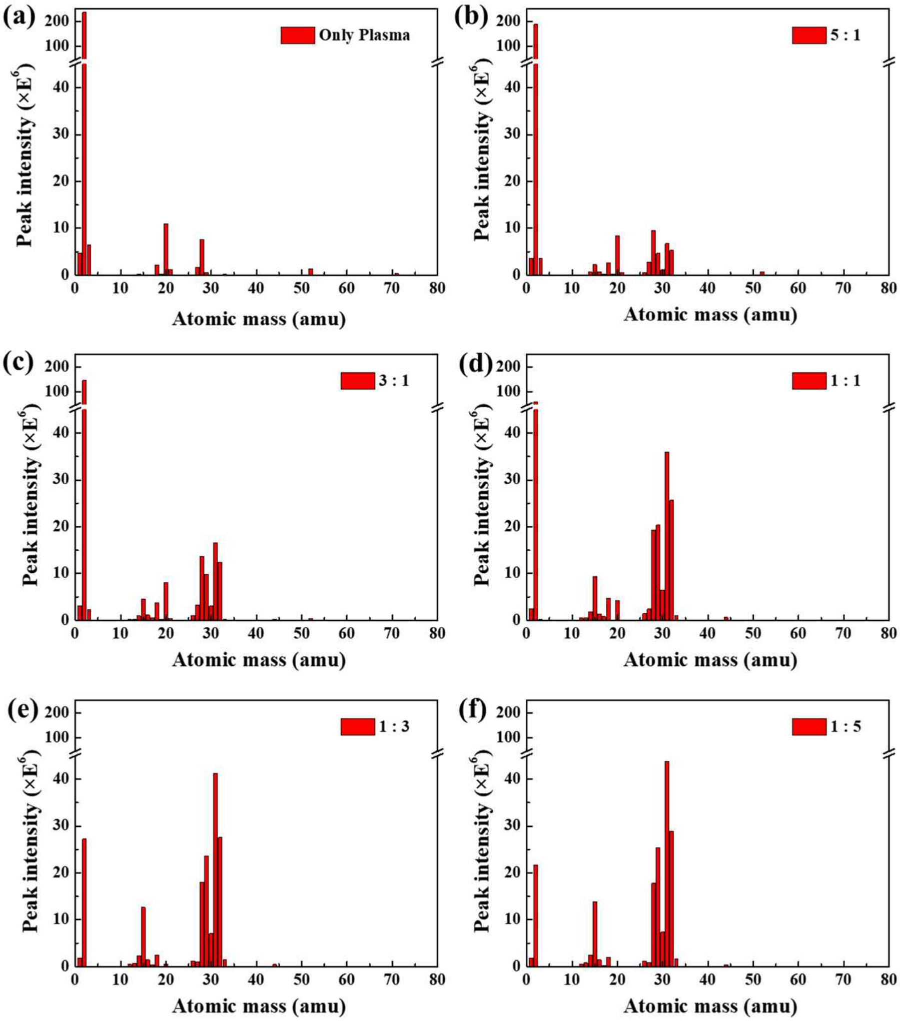

Figure S6. RGA spectra in main chamber region for different NF_3/H_2 : methanol ratios; (a) only plasma condition, (b) 5 : 1 condition, (c) 3 : 1 condition, (d) 1 : 1 condition, (e) 1 : 3 condition, and (f) 1 : 5 condition.

Figure S5 shows RGA spectra in main chamber region for different NF_3/H_2 : methanol ratios.

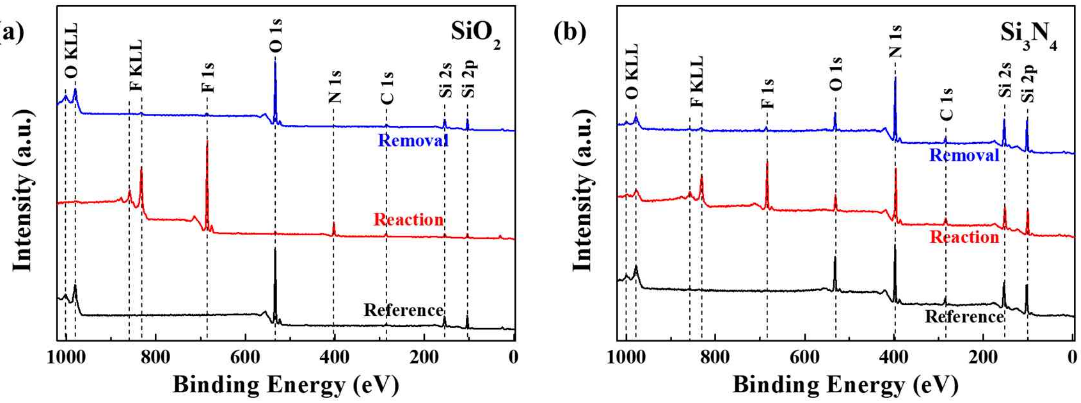

Figure S7. Wide scan XPS data on the surfaces of (a) SiO_2 and (b) Si_3N_4 for each process step of cyclic etching under optimized conditions in Figure 3.

Figure S6 shows the wide scan XPS data for results in Figure 8. As shown in Figure S6(a), on the SiO_2 surface, after the reaction step, F and N peaks were emerged and, after the removal step, those peaks were removed in addition to the increase of O peak. In the case of Si_3N_4, as shown in Figure S6(b), the change of F after each process step similar to that on SiO_2 surface was observed on the Si_3N_4 surface. [12-15]

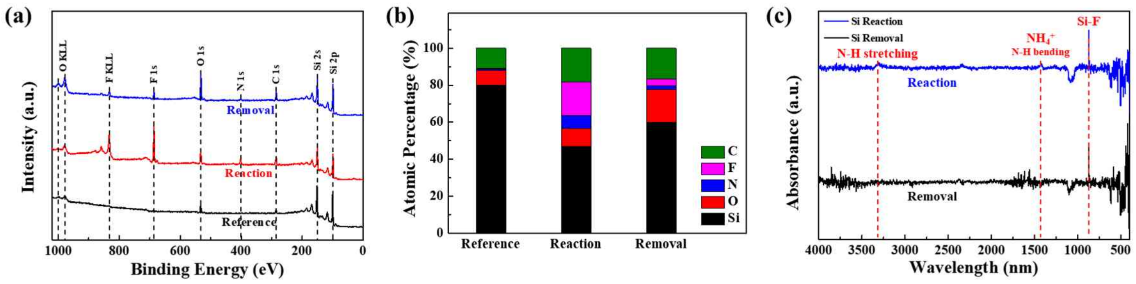

Figure S8. surface characteristics of poly Si measured by XPS and FTIR for each process step of cyclic etching under optimized conditions in Figure 3; (a) XPS wide scan data, (b) surface atomic percentages from (a), and (c) FTIR data.

Figure S7 shows surface characteristics of poly Si measured by XPS and FTIR for each process step of cyclic etching under optimized conditions in Figure 3. As shown in XPS results in Figure S7(a) and (b), the increase of N, O, and F peaks was observed after the reaction step, and, after the removal step, the decrease of N and F peaks was observed but with the increase of O peak possibly due to the oxidation of poly-silicon surface. The FTIR results in Figure S7(c) also showed the slight increase of N-H bonding after the reaction step.

Supporting References

[1] D. H. Kim, J. E. Choi, S. J. Hong, Analysis of optical emission spectroscopy data during silicon etching in $SF_6/O_2/Ar$ plasma, Plasma Sci. Technol. 23 (2021), 125501. https://doi.org/10.1088/2058-6272/ac24f4

[2] Q. Lou, S. Kaler, V. M. Donnelly, D. J. Economou, Optical emission spectroscopic studies and comparisons of CH_3F/CO_2 and CH_3F/O_2 inductively coupled plasmas, J. Vac. Sci. Technol. A. 33 (2015), 021305. http://dx.doi.org/10.1116/1.4904213

[3] A. A. Osipov, A. A. Osipov, V. I Berezenko, S. E. Alexandrov, OES diagnostic of SF_6/Ar gas mixture of ICP discharges for $LiNbO_3$ etching, IOP Conf. Ser.: Mater. Sci. Eng. 919 (2020), 022018. https://doi.org/10.1088/1757-899X/919/2/022018

[4] W. S. Song, J. E. Kang, S. J. Hong, Spectroscopic Analysis of CF_4/O_2 Plasma Mixed with N_2 for Si_3N_4 Dry Etching, Coatings, 12 (2022), 1064. https://doi.org/10.3390/coatings12081064

[5] S. An, S. J. Hong, Spectroscopic Analysis of NF_3 Plasmas with Oxygen Additive for PECVD Chamber Cleaning, Coatings, 13 (2023), 91. https://doi.org/10.3390/coatings13010091

[6] S. J. Kang, V. M. Donnelly, Optical absorption and emission spectroscopy studies of ammonia-containing plasmas, Plasma Sources Sci. Technol. 16 (2007), 265-272. https://doi.org/10.1088/0963-0252/16/2/008

[7] J. Lee, K. Kim, Y. S. Kim, A Study on the NF_3 Plasma Etching Reaction with Cobalt Oxide Grown on Inconel Base Metal Surface, Plasma. Chem. Plasma. Process. 39 (2019), 1145–1159. https://doi.org/10.1007/s11090-019-09979-4

[8] H. W. Liu, T. H. Chen, C. H. Chang, S. K. Lu, Y. C. Lin, D. S. Liu, Impact on the Gas Barrier Property of Silicon Oxide Films Prepared by Tetramethylsilane-Based PECVD Incorporating with Ammonia, Appl. Sci. 7 (2017), 56. https://doi.org/10.3390/app7010056

[9] S. Zhang, X. Zeng, H. Bai, C. Zhang, T. Shao, Optical emission spectroscopy measurement of plasma parameters in a nanosecond pulsed spark discharge for

CO_2/CH_4 dry reforming, Spectrochim. Acta A Mol. Biomol. Spectrosc. 267 (2022), 120590. https://doi.org/10.1016/j.saa.2021.120590

[10] S. Yoo, D. Seok, Y. Jung, K. Lee, Hydrophilic Surface Treatment of Carbon Powder Using CO_2 Plasma Activated Gas. Coatings. 11 (2021), 925. https://doi.org/10.3390/coatings11080925

[11] H. C. Hsueh, H. C. Li, D. Chiang, S. Lee, Effects of Ammonia/Methane Mixtures on Characteristics of Plasma Enhanced Chemical Vapor Deposition n-Type Carbon Films, J. Electrochem. Soc. 159 (2011), D77. https://doi.org/10.1149/2.051202jes

[12] B. Liu, W. Xu, J. Tao, P. Yan, J. Zheng, M. H. Engelhard, D. Lu, C. Wang, J. G. Zhang, Enhanced Cyclability of Lithium–Oxygen Batteries with Electrodes Protected by Surface Films Induced via In Situ Electrochemical Process, Adv. Energy Mater. 8 (2018), 1702340. https://doi.org/10.1002/aenm.201702340

[13] R. Dalmau, R. Collazo, S. Mita, Z. Sitar, X-Ray Photoelectron Spectroscopy Characterization of Aluminum Nitride Surface Oxides: Thermal and Hydrothermal Evolution. J. Electron. Mater. 36 (2007), 414–419. https://doi.org/10.1007/s11664-006-0044-x

[14] M. Fantauzzi, B. Elsener, D. Atzei, A. Rigoldiab, A. Rossi, Exploiting XPS for the identification of sulfides and polysulfides, RSC Adv. 5 (2015), 75953. https://doi.org/10.1039/c5ra14915k

[15] K. T. Wang, W. Y. Wang, T. C. Wei, Photomask-Free, Direct Selective Electroless Deposition on Glass by Controlling Surface Hydrophilicity, ACS Omega, 4 (2019), 7706−7710. http://pubs.acs.org/journal/acsodf
